# Serogroup W135 Meningococcal Meningitis, Northern Cameroon, 2007–2008

**DOI:** 10.3201/eid1502.080988

**Published:** 2009-02

**Authors:** Denis Massenet, Jermias Inrombe, Dave-Etienne Mevoula, Pierre Nicolas

**Affiliations:** Centre Pasteur du Cameroun, Garoua, Cameroon (D. Massenet); Délégation de la Santé du Nord, Garoua (J. Inrombe); Délégation de la Santé de l'Extrême-Nord, Maroua, Cameroon (D.-E. Mevoula); Institut de Médecine Tropicale du Service de Santé des Armées, Marseilles, France (P. Nicolas)

**Keywords:** Neisseria meningitidis serogroup W135, meningitis epidemiology, surveillance, Cameroon, dispatch

## Abstract

We analyzed results of recent microbiologic surveillance of meningitis in northern Cameroon. During the 2007 and 2008 meningitis seasons, all 57 identified meningococcal isolates were serogroup W135. This situation might indicate that the area is experiencing a period between epidemic waves due to 2 different clones of serogroup A meningococci.

The 3 provinces constituting the Septentrion, in North Cameroon, belong to the so-called African meningitis belt (*1*). Until now, in these provinces, the diagnosis of meningitis was made essentially on the basis of clinical signs, and biologic confirmation was uncommon. The rare data that documented meningococci circulating in recent years in this area refer mostly to the early 1990s (*2**,**3*), when serogroup A was by far the most frequently identified serogroup in Cameroon, with strains having the antigenic formula A:4:P1.9 and belonging to the sequence type 5 (ST-5) complex (*4*). From 1999 through 2001, a few cases of meningitis were attributed to serogroup W135 (W135:2a:P1.5,2; ST-11), but these data were from Yaounde, in the southern part of the country (*5*).

## The Study

In 2007, microbiologic surveillance of bacterial meningitis was reintroduced in northern Cameroon to monitor the changing epidemiology of meningococci, with particular attention to uncommon serogroups, such as W135 or X, which have been unusually frequent in Sahelian Africa since 2000 (*6**–**10*). Health authorities decided that an aliquot of every cerebrospinal fluid (CSF) specimen collected in this area would be sent to the Centre Pasteur du Cameroun in Garoua (CPCAG) for testing. Laboratory procedures included assessment of CSF turbidity, Gram staining, search for soluble capsular antigens by using the Pastorex (Bio-Rad, Hercules, CA, USA) latex agglutination kit and dipstick rapid diagnostic test for *Neisseria meningitidis* serogroups A, C, W135, and Y (kindly provided by the Centre de Recherche Médicale et Sanitaire, Niamey, Niger) (*11*). CSF specimens were cultured on blood agar and chocolate agar, supplemented with Polyvitex (bioMérieux, Marcy-l’Etoile, France), with incubation at 37°C with 5% CO_2_. Susceptibility to β-lactam antimicrobial drugs and chloramphenicol was tested according to the recommendations of the Antibiogram Committee of the French Society for Microbiology. The isolates of *N. meningitidis* were sent to the World Health Organization Collaborating Centre (WHOCC) for Reference and Research on Meningococci in Marseilles, France.

Overall, 409 CSF specimens were tested at CPCAG microbiology laboratory from January 1, 2007, through June 30, 2008, of which 144 (35.2%) had a leukocyte count evocative of bacterial meningitis. Appendix Figure 1 shows the monthly distribution of CSF specimens tested at CPCAG.

The number of CSF specimens tested at CPCAG increased greatly after 2006 as the number of health facilities sending specimens for testing increased (Table). This rise was not related to any epidemic, but showed the interest of healthcare workers in microbiologic surveillance of meningitis. The number of tested CSF specimens peaked in March and April, during the dry season, and was low during the rainy season (August and September). Overall, 24 *Streptococcus pneumoniae*, 23 *Haemophilus influenzae*, and 57 *N. meningitidis* isolates were identified, either from culture or by soluble antigens identification.

**Table:** CSF specimens from Cameroon tested at CPCAG laboratory in 2007 and 2008 and number of health facilities that sent specimens, compared with 2006 efforts*

Characteristic	2006	2007	2008†
No. tested CSF specimens	60	202	207
Health facilities involved	Garoua Hospital	Garoua Hospital plus 8 other health facilities	Garoua Hospital plus 17 other health facilities
Specimens not collected in Garoua	0	27	79

*CSF, cerebrospinal fluid; CPCAG, Centre Pasteur du Cameroun in Garoua. †Through June 30.

*S. pneumoniae* and *H. influenzae* were identified throughout the year, but *N. meningitidis* was observed only during the dry season, considered the classical meningitis season in the African meningitis belt (*1*). The median ages of 11 patients with confirmed *S. pneumoniae* infection and 11 patients with confirmed *H. influenzae* infection were 14 and 0.6 years, respectively. Neither *S. pneumoniae* (*12*) nor *H. influenzae* isolates (*8*) were serotyped. The median age of 43 patients with confirmed meningococcal meningitis was 8 years (25th percentile 4.8 years, 75th percentile 15.5 years).

Fifteen *N. meningitidis* isolates were recovered from culture, representing 28.3% of CSF specimens testing positive for soluble antigens. The frequent implementation of early presumptive treatment with antimicrobial drugs, as well as the unsuitable conditions for CSF specimens transport from health facilities, might account for this low rate of positive cultures. The implementation of PCR for the diagnosis of *N. meningitidis*, *S. pneumoniae,* and *H. influenzae* in CPCAG in Garoua in 2009 will enable further enhancement of the surveillance.

All diagnosed meningococci belonged to serogroup W135, although serogroup A has been classically the most prevalent in this Sahelian region, according to surveys carried out in Garoua (*2**,**3*). *N. meningitidis* serogroup A seems to have completely disappeared since the renewal of the microbiologic surveillance of meningitis in northern Cameroon began in February 2007. To our knowledge, there was no previous report in the literature of such a predominance of serogroup W135 in countries of the meningitis belt.

Nine of our 15 *N. meningitidis* isolates were genotyped by using multilocus sequence typing by the WHOCC in Marseille. Eight were identified as ST-2881 and 1 as ST-11. These findings are consistent with the presence of only sporadic cases of serogroup W135 meningococcal meningitis. Until now, ST-2881 has only been associated with sporadic cases or small epidemics, contrary to the findings for ST-11, which was involved in a large epidemic in Burkina Faso in 2002 (*10**,**12*).

We did not observe any resistance of *N. meningitidis* to β-lactam antimicrobial agents and chloramphenicol. These findings did not question probabilistic treatment of suspected cases of meningococcal meningitis based on ceftriaxone or oily chloramphenicol in epidemic situations (*13*).

Laboratory-confirmed cases of serogroup W135 meningococcal meningitis were from Garoua and 9 other health facilities (Appendix Figure 2). Most cases were from the North and Extreme North Provinces. Adamaoua Province seemed to be less affected, likely because of climate and lower population density.

Our results differ clearly from those observed in Yaounde, the capital, which has a subequatorial climate. In 1999–2000, *S. pneumoniae* was the main causative bacterial agent identified in meningitis (109/194, 56%), followed by *H. influenzae* (36/194, 18.6%) and *N. meningitidis* (26/194, 13.4%) (*14*).

WHO does not recommend systematic preventive vaccination with the bivalent A + C polysaccharide vaccine, but does recommend reactive mass immunizations of at-risk populations when the epidemic threshold is crossed. This policy was applied in northern Cameroon in 1992, 1993, 1996, and 1998, when >400,000 doses of vaccine were administered each year for epidemic control. After this period, 30,000–40,000 persons received preventive immunization against *N. meningitidis* serogroups A and C each year. However, the vaccination coverage is too low in this population of ≈6 million inhabitants to explain by itself the current absence of serogroup A meningococci in this area.

## Conclusions

The renewal of microbiologic surveillance of meningitis highlighted the fact that serogroup W135 was the only serogroup of *N. meningitidis* involved in endemic meningitis cases in northern Cameroon in 2007 and 2008. Serogroup A meningococci seemed to be completely absent during this period. This finding might reflect the transitory period separating 2 epidemic waves caused by 2 different clones of serogroup A meningococci (*15*); the emergence of the next epidemic clone is thus possible at any moment. In the meantime, this situation has been considered by WHO, and vaccines that include protection against serogroup W135, such as trivalent vaccine (ACW135), are available for immunization campaigns to control epidemics. This vaccine is available in small quantities and can be delivered only by the International Coordinating Group (created in 1997 by WHO to distribute vaccine ACW135). WHO does not recommend this vaccine when meningococcal meningitis is endemic. Unless an epidemiologic upheaval occurs, future large epidemics of meningococcal meningitis will likely be caused by serogroup A, and the serogroup A conjugate vaccine (which will soon be introduced in the meningitis belt) will be a major advance. However, its successful implementation might create a favorable climate for other serogroups such as W135. In the future, a low-cost conjugate vaccine that includesW135 and perhaps X and Y serogroups can be anticipated.

In the early 2000s, scientists and WHO expressed fear of epidemics caused by serogroup W135 in countries of the African meningitis belt, but the epidemic in Burkina Faso remained isolated. Because the W135 serogroup ST-2881 is known to be less epidemic than W135 ST-11, ST-2881 could have replaced it in the ecologic niche and also have resulted in immunologic protection against ST-11. The observations made in northern Cameroon confirm that we must continue to take into account serogroup W135 and that laboratories should be able to identify it routinely.

## Supplementary Material

 Appendix Figure 1 Monthly distribution of cerebrospinal fluid (CSF) specimens tested in Centre Pasteur du Cameroun in Garoua and identified pathogens (January 2007-June 2008). Black, Neisseria meningitidis; gray, Haemophilus influenzae; hatched, Streptococcus pneumoniae; dotted, turbid CSF without identified etiologic agent; white, crystal clear CSF; line, number of notified cases.

Appendix Figure 2 Map of Cameroon showing the geographic distribution of 53 laboratory-confirmed cases of serogroup W135 meningococcal meningitis (2007-2008). 1, Extreme North Province; 2, North Province; 3, Adamaoua Province; dashed line, southern limit of the African meningitis belt. The number of confirmed cases in a given place is indicated in parentheses. Eq. Guinea, Equatorial Guinea.
