# Supplementary figures and images for: Serogroup W135 Meningococcal Meningitis, Northern Cameroon, 2007–2008
Source: Emerg Infect Dis. 2009 Feb;15(2):340–2. doi: 10.3201/eid1502.080988 (PMC2662656; doi:10.3201/eid1502.080988)

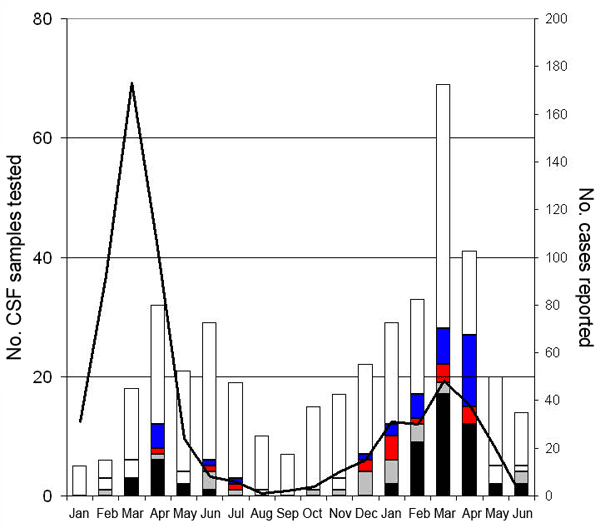

Supplement: Appendix Figure 1 — Monthly distribution of cerebrospinal fluid (CSF) specimens tested in Centre Pasteur du Cameroun in Garoua and identified pathogens (January 2007-June 2008). Black, Neisseria meningitidis; gray, Haemophilus influenzae; hatched, Streptococcus pneumoniae; dotted, turbid CSF without identified etiologic agent; white, crystal clear CSF; line, number of notified cases. [file 08-0988_app1-s1.gif]

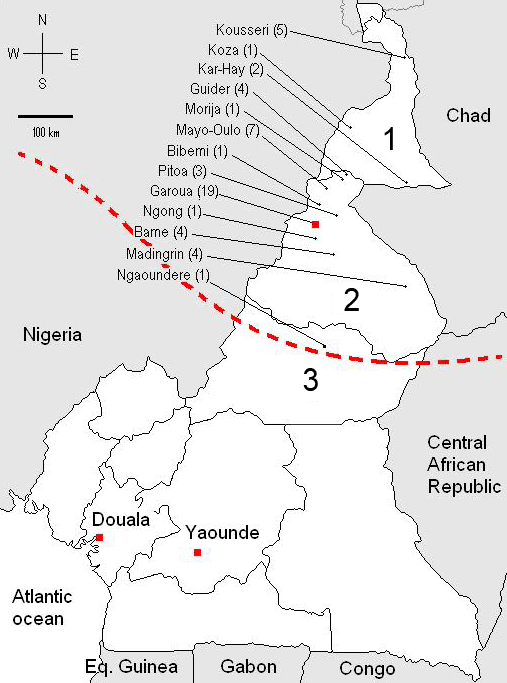

Supplement: Appendix Figure 2 — Map of Cameroon showing the geographic distribution of 53 laboratory-confirmed cases of serogroup W135 meningococcal meningitis (2007-2008). 1, Extreme North Province; 2, North Province; 3, Adamaoua Province; dashed line, southern limit of the African meningitis belt. The number of confirmed cases in a given place is indicated in parentheses. Eq. Guinea, Equatorial Guinea. [file 08-0988_app2-s2.gif]
